# Supplementary material for: Possible poriferan body fossils in early Neoproterozoic microbial reefs
Source: Nature. 2021 Jul 28;596(7870):87–91. doi: 10.1038/s41586-021-03773-z (PMC8338550; doi:10.1038/s41586-021-03773-z)
Supplement: Supplementary file 1 — Reporting Summary [file 41586_2021_3773_MOESM1_ESM.pdf]

nature research

Corresponding author(s): Elizabeth C. Turner
Last updated by author(s): Jun 18, 2021

Reporting Summary

Nature Research wishes to improve the reproducibility of the work that we publish. This form provides structure for consistency and transparency in reporting. For further information on Nature Research policies, see Authors & Referees and the Editorial Policy Checklist.

Statistics

For all statistical analyses, confirm that the following items are present in the figure legend, table legend, main text, or Methods section.

n/a	Confirmed	
☒	☐	The exact sample size (*n*) for each experimental group/condition, given as a discrete number and unit of measurement
☒	☐	A statement on whether measurements were taken from distinct samples or whether the same sample was measured repeatedly
☒	☐	The statistical test(s) used AND whether they are one- or two-sided<br>*Only common tests should be described solely by name; describe more complex techniques in the Methods section.*
☒	☐	A description of all covariates tested
☒	☐	A description of any assumptions or corrections, such as tests of normality and adjustment for multiple comparisons
☒	☐	A full description of the statistical parameters including central tendency (e.g. means) or other basic estimates (e.g. regression coefficient) AND variation (e.g. standard deviation) or associated estimates of uncertainty (e.g. confidence intervals)
☒	☐	For null hypothesis testing, the test statistic (e.g. *F*, *t*, *r*) with confidence intervals, effect sizes, degrees of freedom and *P* value noted<br>*Give P values as exact values whenever suitable.*
☒	☐	For Bayesian analysis, information on the choice of priors and Markov chain Monte Carlo settings
☒	☐	For hierarchical and complex designs, identification of the appropriate level for tests and full reporting of outcomes
☒	☐	Estimates of effect sizes (e.g. Cohen's *d*, Pearson's *r*), indicating how they were calculated

Our web collection on statistics for biologists contains articles on many of the points above.

Software and code

Policy information about availability of computer code

Data collection	Photomicrographs were imaged using Luminera Infinity Analyze v.6.5 and Qcapture 2.98.0.
Data analysis	No software was used for data analysis.

For manuscripts utilizing custom algorithms or software that are central to the research but not yet described in published literature, software must be made available to editors/reviewers. We strongly encourage code deposition in a community repository (e.g. GitHub). See the Nature Research guidelines for submitting code & software for further information.

Data

Policy information about availability of data

All manuscripts must include a data availability statement. This statement should provide the following information, where applicable:

- Accession codes, unique identifiers, or web links for publicly available datasets
- A list of figures that have associated raw data
- A description of any restrictions on data availability

No databases were generated in or used by this study.

Field-specific reporting

Please select the one below that is the best fit for your research. If you are not sure, read the appropriate sections before making your selection.

☐ Life sciences ☐ Behavioural & social sciences ☒ Ecological, evolutionary & environmental sciences

For a reference copy of the document with all sections, see nature.com/documents/nr-reporting-summary-flat.pdf

Ecological, evolutionary & environmental sciences study design

All studies must disclose on these points even when the disclosure is negative.

Study description	Describes petrographic evidence of possible body fossils of sponges that are approximately 890 million years old.
Research sample	Thin sections (30-micon-thick rock slices) from fossil reef rock in the Stone Knife Formation ("Little Dal reefs") in Northwest Territories, Canada.
Sampling strategy	Over a thousand approximately fist-sized rock samples were separated from natural rock exposures using a rock hammer. The samples were later slabbed and thin-sectioned. Initially, samples had been collected for an unrelated purpose (documenting reef microbialites in a separate, published study); sample distribution was randomly dispersed throughout all exposed parts of selected reefs in an attempt capture spatial variability in microbialites, which are not discernible on natural rock exposures. Sample field locations were documented using photographs and diagrams; obtaining accurate GPS points for sample locations is not possible given the extreme topography (limits satellite access) and the small size of the samples relative to GPS error. Areas where the thin sections contained vermiform microstructure were later revisited and resampled. Sample size is considerably larger than the masses of vermiform microstructure. Sample distribution in the reefs is dense enough and reef facies well enough understood (previously published study) for the reefal subenvironments in which vermiform microstructure is preserved to be characterised.
Data collection	Rock samples were separated from natural rock exposures using a rock hammer. Samples were shipped to the lab, sawed, polished, and thin-sectioned using standard petrographic preparation.
Timing and spatial scale	Rock samples were collected during field work between 1992 and 2018. Sample size is considerably larger than the masses of vermiform microstructure that are the subject of the study.
Data exclusions	No data were excluded.
Reproducibility	Reproducibility depends on locating the exact field locations and places on exposure surfaces from which samples containing vermiform microstructure were collected. Field locations of rock samples were recorded in detail using photographs and diagrams. Revisiting and resampling these locations in the years following the initial collection successfully yielded more material containing vermiform microstructure in thin section.
Randomization	This was not an experimental study.
Blinding	This was not an experimental study.

Did the study involve field work? ☒ Yes ☐ No

Field work, collection and transport

Field conditions	Remote alpine-subarctic locations in the Mackenzie Mountains, Northwest Territories, Canada, that are accessible only by helicopter. Field work is possible only in the summer months when snow cover is minimal (mid-June to mid-August).
Location	Numerous locations between 64°47’N / 129°35’W and 64°59’N / 130°55’W.
Access and import/export	Field work was conducted under science licencing from the Aurora Research Institute (2016 Licence #15888; 2017, 2018 Licence #15993), and associated permissions from land use, water board, renewable resource, community, land claim, band council, and hunting-trapping organisations.
Disturbance	No temporary nor long-term disturbances were introduced.

Reporting for specific materials, systems and methods

We require information from authors about some types of materials, experimental systems and methods used in many studies. Here, indicate whether each material, system or method listed is relevant to your study. If you are not sure if a list item applies to your research, read the appropriate section before selecting a response.

Materials & experimental systems

n/a	Involved in the study	
☒	☐	Antibodies
☒	☐	Eukaryotic cell lines
☐	☒	Palaeontology
☒	☐	Animals and other organisms
☒	☐	Human research participants
☒	☐	Clinical data

Methods

n/a	Involved in the study	
☒	☐	ChIP-seq
☒	☐	Flow cytometry
☒	☐	MRI-based neuroimaging

Palaeontology

Specimen provenance	Field work was conducted under science licencing from the Aurora Research Institute (2016 Licence #15888; 2017, 2018 Licence #15993), and applicable associated permissions from land use, water board, renewable resource, community, land claim, band council, and hunting-trapping organisations.
Specimen deposition	Field data, rock samples, and thin sections are archived in the author's collection at Laurentian University.
Dating methods	No new dates are presented.

☐ Tick this box to confirm that the raw and calibrated dates are available in the paper or in Supplementary Information.
